# Comparative analysis of the sexual function of Nigerians with type 2 diabetes and apparently healthy controls

**DOI:** 10.4314/gmj.v59i4.10

**Published:** 2025-12

**Authors:** Olufemi O Oyewole, Ayotunde O Ale, Ayanbola I Adepoju, Grace M Emmanuel, Olatunde Odusan

**Affiliations:** 1 Physiotherapy Department, Olabisi Onabanjo University Teaching Hospital, Sagamu, Nigeria; 2 Medicine Department, Olabisi Onabanjo University Teaching Hospital, Sagamu, Nigeria; 3 College of Health Sciences, University of KwaZulu-Natal, Private Bag X54001, Durban, South Africa

**Keywords:** Sexual functioning, Sexual dysfunction, Prevalence, Type 2 diabetes mellitus, Nigeria

## Abstract

**Objective:**

To assess the sexual functioning of Nigerians with T2DM in comparison to healthy controls.

**Design:**

Comparative descriptive cross-sectional survey.

**Setting:**

Olabisi Onabanjo University Teaching Hospital.

**Participants:**

A Consecutive sample of 210 adult Nigerians with T2DM and 121 without diabetes.

**Main outcome measures:**

Sexual functioning was assessed with the Changes in Sexual Functioning Questionnaire.

**Results:**

Participants with diabetes had a higher prevalence of SD than those without diabetes (76.2% vs 34.7%). This remained unchanged when stratified by gender and sexual response cycles. Increasing age and female gender were significantly associated with SD among participants with diabetes. A one-year increase in age has 1.09 odds of increased SD among participants with diabetes (OR = 1.09, CI = 1.05 – 1.13), while the female participants demonstrated higher SD when compared with males, irrespective of diabetes status, [with diabetes (80.7% vs 65%) and without diabetes (60% vs 20.6%)]. They were also three times as likely to have SD as their male counterparts (OR = 3.39, CI = 1.59–7.24, p = 0.002).

**Conclusion:**

People with diabetes reported lower sexual functioning compared with those without diabetes, with a more than double the prevalence. Females had a higher prevalence of SD than males, irrespective of diabetes status, associated with age among participants with diabetes.

**Funding:**

None declared

## Introduction

Diabetes mellitus is a disease of global health concern with many systemic chronic complications, of which sexual dysfunction is one. Sexual dysfunction is commonly reported among patients with diabetes. It includes erectile dysfunction, premature ejaculation, low libido, hypoactive sexual desire disorder, lack of sexual satisfaction, low vaginal lubrication, and orgasmic dysfunction.^1^–^6^ Sexual dysfunction often leads to psychological disturbances affecting relationships with a negative impact on family life and social activities.^2^,^3^

A review of the literature suggests a high prevalence of sexual dysfunction among patients with diabetes compared with those without diabetes.^7^–^10^ Sexual dysfunction among patients with diabetes was two to four times higher when compared with those without diabetes.^7^,^11^

Study findings from Nigeria suggest that sexual dysfunction was more prevalent among patients with diabetes compared with those without diabetes.^8^,^12^ In northwest and southwest Ethiopia, the prevalence of sexual dysfunction of 69.5% and 53.3% was reported among patients with diabetes, respectively.^6^,^13^ The reported prevalence of erectile dysfunction among patients with diabetes was as high as 94.7% among Nigerian men with diabetes 14, 81.1% in eastern Sudan 4, 80% in Egypt 15, 64.2% in Chinese men 16, 60.2% in Bangladeshi men^17^, and 59.4% in India.^18^ In comparison, female sexual dysfunction of 88.7% was reported in Saudi women^1^, 72% in the Philippines^19^, 70% in the Netherlands^5^, 64.5% in Pakistan^3^, and 55.9% in Turkish women with diabetes.^20^ Meta-analysis and systematic review indicate 68.6% overall prevalence of sexual dysfunction among female patients with diabetes^21^,^22^ while 71.5% erectile dysfunction among male patients with diabetes was reported.^21^,^22^ Erectile dysfunction was about four times as high among men with diabetes compared with those without diabetes.^9^ The variability rates in sexual function reported in the aforementioned studies may be due to the age and gender differences of the study participants.

Several risk factors have been implicated in the high prevalence of sexual dysfunction among patients with diabetes. Among these risk factors are older age, physical inactivity, depression, menstrual cycle phases, and menopause.^1^,^3^,^6^,^8^,^12^,^13^,^17^,^23^ Other determinants of sexual dysfunction among patients with diabetes include longer duration of diabetes, poor glycemic control, albuminuria, obesity, complications of neuropathy, coronary artery disease, as well as cardiovascular and neurological comorbidities.^13^,^15^,^16^,^18^–^20^,^24^,^25^ The potential mechanisms by which the diagnosis of diabetes leads to sexual dysfunction have been attributed to the presence of these risk factors, especially vascular, neuropathic and hormonal complications of diabetes.^26^ It is therefore essential to regularly assess sexual functioning among patients with diabetes, demonstrating these characteristics for optimum care.

Most often, patients with diabetes are not asked or assessed for sexual dysfunction during clinic attendance.^27^ This is attributable to the unwillingness of many of such patients to voluntarily express sexual dysfunction to healthcare providers.^2^ Reluctance to express sexual dysfunction is attributed to stigma, associated with low self-esteem, and a busy clinic environment^2^ highlighting the need for regular assessment of sexual functioning among this population. In Nigeria, like other African countries, about 75% of patients with diabetes have poor sexual health-seeking behaviour.^28^ Anecdotal reports suggest that cultural influence does not encourage Nigerians to freely discuss sexual dysfunction, as this is often seen as a sign of promiscuity, especially among women. Few studies that assessed sexual dysfunction in Nigeria focused on specific domains of sexual dysfunction and are gender specific. Therefore, these studies could not highlight the precise burden or provide a combined overview of both genders.^8^,^12^,^14^ In Nigeria, there is a paucity of literature comparing sexual functioning among both genders in people with diabetes. Using a generic measure of sexual function, information to bridge this gap in knowledge may be provided. This study sought to assess the sexual functioning status of Nigerians with type 2 diabetes mellitus and compare it with that of those without diabetes.

## Methods

### Study design

A comparative, cross-sectional analysis of sexual functioning among Nigerians with Type 2 DM and apparently healthy controls.

### Study population

Adult Nigerians who were 25 years and above with a diagnosis of type 2 diabetes mellitus, using the World Health Organisation (WHO)^29^ criteria and attending the diabetes clinic of Olabisi Onabanjo University Teaching Hospital, and who gave informed consent were consecutively recruited. Gender and age-matched apparently healthy controls were recruited among the staff of the tertiary hospital and from the community where the participants with diabetes lived. Preceding the study, Fischer's formula was used to determine the sample size for participants with diabetes, whereby at least 199 participants with diabetes are needed to power the study based on a Confidence Level of 95%, p = 0.5, Error (Margin) of 0.05, Z-score = 1.959964, and population of attendees at the diabetic clinic of 414.^30^ Cohen's table was used to estimate the sample size for apparently healthy controls, assuming an effect size of 0.50 for sexual dysfunction between participants with diabetes and apparently healthy controls, with a significance level of 0.05 and 95% power.^31^ Therefore, a minimum of 89 apparently healthy controls is required for the study.

### Ethical consideration

Olabisi Onabanjo University Teaching Hospital Health Research Ethics Committee approved the study protocol (Approval no: OOUTH/HREC/472/2021AP). The participants provided written informed consent.

### Measurement

The ‘Changes in Sexual Functioning Questionnaire’ (CSFQ-14) was used to assess the sexual functioning of study participants. CSFQ-14 is a 14-item questionnaire on a 5-point Likert scale ranging from ‘never’ (one) to ‘every day/always’ (five). Questions 10 and 14 were negatively worded and were reversed during analysis. The CSFQ-14 has been validated among patients with diabetes and healthy participants and adjudged valid even among Nigerian patients, with a Cronbach's alpha ranging between 0.72 and 0.90, while it is 0.91in the present study.^6^,^32,33^ The CSFQ-14 measures five types of sexual function: pleasure, desire/frequency, desire/interest, arousal/erection, and orgasm/ejaculation which allows two separate schemas: three scales domain [Desire (items 2–6), Arousal (items 7–9), and Orgasm or Completion (items 11–13)] corresponding to the phases of the sexual response cycle; and five scales domain [Pleasure (item 1)], [Desire/Frequency (items 2–3),

Desire/Interest (items 4–6), Arousal/Excitement (items 7–9), and Orgasm/Completion (items 11–13)]. Items 10 and 14 were not included in any schemas but were included in the total scores of sexual functioning, which range between 14 and 70. The threshold scores used to define sexual dysfunction were pleasure phase <4, desire/Frequency phase <6, desire/Interest phase <9, desire phase <15, arousal phase <12, orgasm phase <11, and global dysfunction <41.^33^

### Statistical analysis

Missing data were replaced using the series mean. Data were subjected to normality tests, and sexual functioning data were found not to be normally distributed. Descriptive statistics (mean, median, standard deviation, frequency, percentages) and charts were used to examine the data. Differences in sexual functioning between people with and without diabetes were assessed using the Mann-Whitney U and x^2^ test. Logistic regression analysis was used to examine the associations of sexual dysfunction, and the test of significance, P-values, were set at 0.05.

## Results

Two hundred and ten participants with diabetes (female = 150, male = 60) and 121 without diabetes (female = 58, male = 63) were included in the study, with a mean age of 59.7±11.6 and 52.4±9.4 years (p = 0.0001), respectively. There were no significant age differences between the male and female participants in both groups (p < 0.05).

Study participants' responses to items of CSFQ-14 are shown in Table 1. On a scale of one to five, many of the participants with diabetes never or rarely enjoy their sex life (2.01), nor frequently engage in sexual activity (1.89) and sexual thought (2.08). Participants without diabetes do most of the time or sometimes (2.83 – 3.06). Furthermore, most female participants with diabetes were not easily aroused (1.85) and did not often experience orgasm (1.81), while female participants without diabetes did most of the time or sometimes (2.50 – 3.00). However, many of the study participants (with and without diabetes) do not enjoy books, movies, music, or artwork with sexual content (2.06 vs 2.38) nor experience painful orgasms (4.61 vs 4.38).

**Table 1:** Participants' response distribution on CSFQ-14 scale

Item	Participants with diabetes (N=210, women=150, men=60)						Participants without diabetes (N=121, women=58, men=63)					

	Never n(%)	Rarely n(%)	Sometimes n(%)	Most of the time n(%)	Everyday n(%)	Mean (median)	Never n(%)	Rarely n(%)	Sometime n(%)	Most of the time n(%)	Everyday n(%)	Mean (median)
**Compared with the most enjoyable it has ever been, how enjoyable or pleasurable is your sex life right now?**	102 (48.6)	35 (16.7)	48 (22.9)	19 (9.0)	6 (2.9)	2.01 (2.00)	6 (5.0)	18 (14.9)	64 (52.9)	29 (24.0)	4 (3.3)	3.06 (3.00)
**How frequently do you engage in sexual activity (sexual intercourse, masturbation, etc.) now?**	104 (49.5)	47 (22.4)	40 (19.0)	16 (7.6)	3 (1.4)	1.89 (2.00)	8 (6.6)	18 (14.9)	74 (61.2)	19 (15.7)	2 (1.7)	2.91 (3.00)
**How often do you desire to engage in sexual activity?**	89 (42.4)	40 (19.0)	54 (25.7)	24 (11.4)	3 (1.4)	2.10 (2.00)	8 (6.6)	21 (17.4)	77 (63.6)	13 (10.7)	2 (1.7)	2.83 (3.00)
**How frequently do you engage in sexual thoughts (thinking about sex, sexual fantasies) now?**	86 (41.0)	53 (25.2)	47 (22.4)	16 (7.6)	8 (3.8)	2.08 (2.00)	22 (18.2)	39 (32.2)	49 (40.5)	8 (6.6)	3 (2.5)	2.43 (2.00)
**Do you enjoy books, movies, music or artwork with sexual content?**	96 (45.7)	50 (23.8)	31 (14.8)	22 (10.5)	11 (5.2)	2.06 (2.00)	40 (33.1)	24 (19.8)	35 (28.9)	15 (12.4)	7 (5.8)	2.38 (2.00)
**How much pleasure or enjoyment do you get from thinking about and fantasising about sex?**	96 (45.7)	43 (20.5)	43 (20.5)	18 (8.6)	10 (4.8)	2.06 (2.00)	21 (17.4)	42 (34.7)	42 (34.7)	13 (10.7)	3 (2.5)	2.46 (2.00)
**How often do you become sexually aroused? (Women)**	79 (52.7)	25 (16.7)	37 (24.7)	7 (4.7)	2 (1.3)	1.85 (1.00)	9 (15.5)	16 (27.6)	28 (48.3)	5 (8.6)	0 (0.0)	2.50 (3.00)
**How often do you have an erection related or unrelated to sexual activity? (Men)**	18 (30.0)	23 (38.3)	10 (16.7)	7 (11.7)	2 (3.3)	2.00 (2.00)	2 (3.2)	10 (15.9)	35 (55.6)	7 (11.1)	9 (14.3)	3.17 (3.00)
**Are you easily aroused? (Women)**	82 (54.7)	37 (24.7)	16 (10.7)	11 (7.3)	4 (2.7)	1.79 (1.00)	8 (13.8)	12 (20.7)	24 (41.4)	11 (19.0)	3 (5.2)	2.81 (3.00)
**Do you get an erection easily? (Men)**	18 (30.0)	21 (35.0)	8 (13.3)	7 (11.7)	6 (10.0)	2.37 (2.00)	2 (3.2)	13 (20.6)	13 (20.6)	24 (38.1)	11 (17.5)	3.45 (4.00)
**Do you have adequate vaginal lubrication during sexual activity (get wet)? ((Women)**	87 (58.0)	26 (17.3)	18 (12.0)	13 (8.7)	6 (4.0)	1.83 (1.00)	11 (19.0)	6 (10.3)	21 (36.2)	13 (22.4)	7 (12.1)	2.98 (3.00)
**Are you able to maintain an erection? (Men)**	21 (35.0)	15 (25.0)	13 (21.7)	7 (11.7)	4 (6.7)	2.30 (2.00)	2 (3.2)	12 (19.0)	17 (27.0)	23 (36.5)	9 (14.3)	3.4 (4.00)
**How often do you become aroused and then lose interest? (Women)**	3 (2.0)	10 (6.7)	27 (18.0)	27 (18.0)	83 (55.3)	4.18 (5.00)	2 (3.4)	4 (6.9)	24 (41.4)	17 (29.3)	11 (19.0)	3.53 (3.00)
**How often do you experience painful, prolonged erections? (Men)**	0 (0.0)	1 (1.7)	7 (11.7)	17 (28.3)	35 (58.3)	4.43 (5.00)	0 (0.0)	2 (3.2)	11 (17.5)	8 (12.7)	42 (66.7)	4.43 (5.00)
**How often do you experience an orgasm? (Women)**	85 (56.7)	20 (13.3)	32 (21.3)	12 (8.0)	1 (0.7)	1.81 (1.00)	5 (8.6)	8 (13.8)	31 (53.4)	10 (17.2)	4 (6.9)	3.00 (3.00)
**How often do you have an ejaculation? (Men)**	22 (36.7)	12 (20.0)	18 (30.0)	8 (13.3)	0 (0)	2.20 (2.00)	0 (0)	8 (12.7)	30 (47.6)	21 (33.3)	4 (6.3)	3.33 (3.00)
**Are you able to have an orgasm when you want to? (Women)**	86 (57.3)	23 (15.3)	27 (18.0)	12 (8.0)	2 (1.3)	1.81 (1.00)	10 (17.2)	9 (15.5)	25 (43.1)	7 (12.1)	7 (12.1)	2.86 (3.00)
**Are you able to ejaculate when you want to? (Men)**	25 (41.7)	11 (18.3)	13 (21.7)	11 (18.3)	0 (0.0)	2.17 (2.00)	6 (9.5)	13 (20.6)	16 (25.4)	20 (31.7)	8 (12.7)	3.17 (3.00)
**How much pleasure or enjoyment do you get from your orgasms?**	113 (53.8)	29 (13.8)	38 (18.1)	25 (11.9)	5 (2.4)	1.95 (1.00)	6 (5.0)	15 (12.4)	40 (33.1)	47 (38.8)	13 (10.7)	3.38 (3.00)
**How often do you have painful orgasm?**	2 (1.0)	5 (2.4)	19 (9.0)	20 (9.5)	164 (78.1)	4.61 (5.00)	2 (1.7)	4 (3.3)	17 (14.0)	21 (17.4)	77 (63.6)	4.38 (5.00)

The differences in sexual functioning between the study participants are shown in Table 2. Participants with diabetes demonstrated significantly lower sexual functioning compared with those without diabetes which when stratified by gender, this observation remained the same except for the desire phase among the male participants. Female participants demonstrated significantly lower global sexual functioning compared with male participants in both groups of people. The significantly lower sexual functioning among females was also observed when stratified by the three-scale (3-phase cycle of sexual response) and five-scale domains, except for the desire phase, among participants without diabetes.

**Table 2:** Participants with and without diabetes's sexual functioning

Variable	Participants with diabetes				Participants without diabetes				Comparison between participants with and without diabetes		

									Both gender	Male	Female
	Male X±SD	Female X±SD	*P*	Both gender X±SD	Male X±SD	Female X±SD	*P*	Both gender X±SD	*P*	*P*	*P*
**3-scale domain**											
**Desire**	11.87±4.72	9.53±4.58	<0.001	10.20±4.73	13.37±3.26	12.64±2.59	0.507	13.02±2.97	<0.001	0.072	<0.001
**Arousal**	6.87±3.43	5.47±3.07	0.003	5.87±3.23	10.03±2.48	8.29±2.60	<0.001	9.20±2.67	<0.001	<0.001	<0.001
**Orgasm**	6.68±3.22	5.44±3.10	0.005	5.80±3.18	10.17±2.17	8.93±2.78	0.019	9.58±2.55	<0.001	<0.001	<0.001
**5-scale domain**											
**Pleasure**	2.23±1.14	1.92±1.16	0.035	2.01±1.16	3.21±0.77	2.90±0.91	0.036	3.06±0.85	<0.001	<0.001	<0.001
**Desire/freq.**	4.52±1.86	3.79±2.07	0.006	4.00±2.03	6.00±1.21	5.47±1.35	0.037	5.74±1.30	<0.001	<0.001	<0.001
**Desire/interest**	7.35±3.35	5.74±2.93	<0.001	6.20±3.13	7.37±2.59	7.17±2.07	0.898	7.27±2.35	<0.001	0.844	<0.001
**Arousal**	6.87±3.43	5.47±3.07	0.003	5.87±3.23	10.03±2.48	8.29±2.60	<0.001	9.20±2.67	<0.001	<0.001	<0.001
**Orgasm**	6.68±3.22	5.44±3.10	0.005	5.80±3.18	10.17±2.17	8.93±2.78	0.019	9.58±2.55	<0.001	<0.001	<0.001
**Global dysfunction**	36.67±10.71	31.17±10.01	<0.001	32.74±10.49	45.89±6.74	40.34±6.80	<0.001	43.23±7.29	<0.001	<0.001	<0.001
**Age**	61.78±11.25	58.87±11.65	0.099	59.70±11.59	53.68±10.68	50.93±7.50	0.106	52.36±9.36	<0.001	<0.001	<0.001

**Figure 1:**
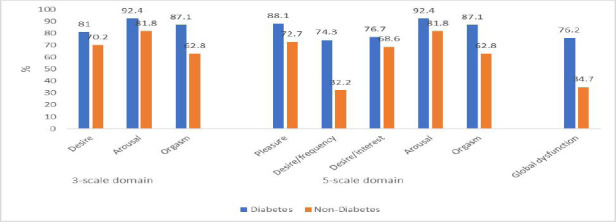
Prevalence of sexual dysfunction among participants with and without diabetes

**Figure 2:**
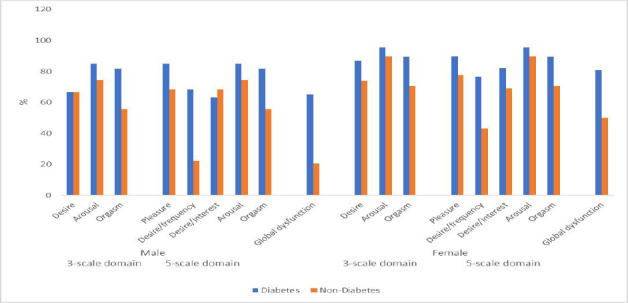
Prevalence of sexual dysfunction among participants with and without diabetes by gender

The prevalence of sexual dysfunction is shown in Figure 1, with the global prevalence of sexual dysfunction of 76.2% and 34.7% among participants with and without diabetes (*χ*^2^ = 55.54; p = 0.001), respectively. In both the 3-scale (3-phase cycle of sexual response) and 5-scale domains, participants with diabetes have a higher proportion of prevalence of sexual dysfunction than participants without diabetes. The same pattern of prevalence of sexual dysfunction was observed when stratified by gender (p = 0.001), with the exception of the desire phase among the male participants (Figure 2).

Table 3 presents the logistic regression results. When age and gender were entered into logistics regression, both were significantly associated with sexual dysfunction among participants with diabetes (Nagelkerke R^2^ = 22.7%), while only gender was significantly associated with sexual dysfunction among participants without diabetes (Nagelkerke R^2^ = 14.5).

**Table 3:** Logistic regression of factors that are associated with sexual dysfunction

Variable	B	S.E.	Wald	*P*	OR	95% C.I. (OR)	

						Lower	Upper
**Participants with diabetes**							
**Constant**	-4.523	1.087	17.324	<0.001	0.011		
**Age (year)**	0.085	0.018	23.522	<0.001	1.089	1.052	1.127
**Gender (ref = male)**	1.220	0.387	9.931	0.002	3.388	1.586	7.237
**Participants without diabetes**							
**Constant**	-2.962	1.300	5.188	0.023	0.052		
**Age (year)**	0.030	0.023	1.693	0.193	1.030	0.985	1.077
**Gender (ref = male)**	1.457	0.424	11.824	<0.001	4.292	1.871	9.847

Female participants with and without diabetes were three and four times more likely to have sexual dysfunction compared with their male counterparts, [OR = 3.39, CI = 1.59 – 7.24, (p = 0.002) Vs OR = 4.29, CI = 1.89 – 9.85, (p = 0.001)] respectively. With a unit increase in age, participants with diabetes have 1.09 odds of sexual dysfunction (OR = 1.09, CI = 1.05 – 1.13).

## Discussion

Our study set out to study sexual functioning among Nigerian individuals with T2 Diabetes and compare it with that of those without diabetes. The study revealed that many participants with diabetes never or rarely enjoy their sex life, infrequently engage in sexual activity and sexual thought, are not easily aroused, nor often experience orgasm, while participants without diabetes do most of the time. The prevalence of sexual dysfunction was more than double among participants with diabetes compared with those without diabetes. Female participants demonstrated consistently higher sexual dysfunction in comparison with their male counterparts.

Findings from this study are consistent with previous studies, which showed that people with diabetes have lower sexual functioning than those without diabetes.^7^,^9^,^10^,^12^ This may suggest that diabetes significantly impacts sexual functioning. Thus, attention should be focused on sexual health or counselling for all people with diabetes with the aim of early diagnosis and treatment of sexual dysfunction among this population. Particular attention should be directed at erectile dysfunction, vaginal lubrication, and orgasm, as our data suggest these to be the most affected.

We observed more than double the prevalence of sexual dysfunction among participants with diabetes compared with those without diabetes. This is in tandem with previous studies, which have reported a 2 – 4 times higher prevalence of sexual dysfunction among people with diabetes.^7^,^9^ Diabetes has been linked to sexual dysfunction through a pathophysiological mechanism that involves insults to the neuronal, vascular, hormonal, and psychological systems caused by hyperglycaemia. The sex hormones (Gonadotropin-releasing hormone, follicle-stimulating hormone, luteinizing hormone, and testosterone) are secreted less frequently in males due to damage to the hypothalamus, pituitary gland, gonads, and perigonads caused by insults such as oxidative stress and abnormal zinc metabolism. Seminiferous tubular damage, spermatogenic cell damage, testicular shrinkage, stromal cell atrophy, and other structural harm to the male reproductive organs can result from them.^34,35^ In contrast, hyperglycaemia may change the hypothalamic-pituitary-gonadal axis, genital neurovascular processes, and nitric oxide/cyclic guanosine monophosphate production in females, resulting in vaginal and clitoris vasocongestion, vaginal dryness, dyspareunia, and reduced ovarian and sexual functionality.^36,37^

Despite the varied instruments used to assess sexual dysfunction among this study population, the prevalence of sexual dysfunction (76.2%) in the present study is similar to the previous studies that reported values ranging from 69% to 81%.^1^,^2^,^4^,^19^,^25^ Two studies were found using the same tool to assess sexual dysfunction as ours.^6^,^13^ The proportion of participants with diabetes who reported sexual dysfunction was higher in the present study than in the report from southwest Ethiopia report^6^ with their male participants reporting a similar rate of sexual dysfunction (65% vs 69.5%).^13^ The present study participants are older, with more female participants than the two previous studies, which used the same tool as ours. Previous studies using the same data suggest that about 34% had peripheral arterial disease 22% had neuropathy, with 28% neuropathic pain.^38,39^ These factors have been associated with higher sexual dysfunction, which may account for the higher rate in the present study. It is worrisome to note that well over three-quarters of participants with diabetes in the present study reported sexual dysfunction, reflecting the impact of diabetes on sexual function. Therefore, people with diabetes should be frequently assessed for sexual dysfunction not only to determine the magnitude but to help those who might be suffering from the problem.

Despite a similarity in sexual desire among the study participants, the mean values of sexual functioning were consistently lower among participants with diabetes compared with those without diabetes. Sexual function has been said to be a cumulative effect of the neurologic, vascular, hormonal, and psychological systems.^2^,^13^,^24^ Diabetes and its complications influence these variables and may account for the observation of low sexual functioning. This is a problem among people with diabetes that cannot be ignored by healthcare providers since they show a similar desire for sex as those without diabetes. Unattended sexual dysfunction may result in dissatisfaction with sex, psychological problems, and marital relationships, culminating in poor quality of life (QoL).^7^ Therefore, diligent screening and prompt treatment are necessary to mitigate the problem.

The present study found gender differences in sexual functioning to be less prevalent in males. This is corroborated by the regression analysis. Female participants (with and without diabetes) were three and four times more likely to have sexual dysfunction compared with their male counterparts. Several reasons may be attributed to higher sexual dysfunction among females in our study. Most women in our study have reached the age of menopause, as this has been linked with decreased sexual functioning among women.^12^,^40^ Others are cultural and religious beliefs. Women do not express sex openly, as it is seen as a sign of promiscuity and propensity to infidelity in many Nigerian populations.^41^ A previous study has reported highly prevalent sexual inactivity among middle-aged and older women with early type 2 diabetes, which was three times more prevalent in women than men.^42^

A further finding from this study was the influence of age on sexual function. Although there was no significant age difference between male and female participants in the present study, the difference in age of participants with and without diabetes may also add to the increased global sexual dysfunction among participants with diabetes. The increased risk of sexual dysfunction at an older age has been attributed to changes in hormonal function, poor glycemic control, increased diabetes-related complications and/or other co-morbidities, and advanced age, which correlated with long duration of treatment, as these often affect sexual function.^6^ Regression analysis revealed that with a unit increase in age, participants with diabetes have increased odds of sexual dysfunction, which is in tandem with previous studies with similar findings.^1^,^6^,^14^

This study has some clinical implications. Given the fact that many people with diabetes do not discuss sexual dysfunction with healthcare providers despite its presence, there should be a routine screening of sexual dysfunction of attendees at diabetic clinics for early identification and prompt treatment^2^,^14^. Though men are more willing to discuss their sexual dysfunction with their healthcare provider than women, both genders should be screened, though healthcare professionals often overlook this important aspect of life.^25^,^27^

The strength of the current study is the usage of a generic tool to assess sexual functioning, which allows for comparison in both genders, and has an apparently healthy control group from the same community. However, study limitations to be considered when interpreting the current results include (a) the cross-sectional nature, for we do not know if the intervention (i.e. the treatment received by people with diabetes) could modify the prevalence of sexual dysfunction. Furthermore, (b) participants may under-report the sexual dysfunction as the study was a culturally sensitive and embarrassing issue; therefore, social desirability bias could not be ruled out, and (c) control participants were selected based on self-reported health status. Chronic health conditions may be present and can only be identified through thorough screening. The data were from a single institution, and generalizability to the general population may not be applicable. Despite these limitations, we believe our study has shed more light on this sensitive issue among Nigerians with diabetes, as well as established a comparison between people with and without diabetes in Nigeria, which is important within the healthcare system.

## Conclusion

People with diabetes reported lower sexual functioning in comparison with those without diabetes. The prevalence of sexual dysfunction was more than double among diabetic participants. Female participants demonstrated consistently higher sexual dysfunction compared with males. Regular screening and sexual health counselling are therefore recommended in all diabetes clinics for the identification and prompt management of sexual dysfunction.
